# Aerobic Mesophilic, Coliform, *Escherichia coli*, and *Staphylococcus aureus* Counts of Raw Meat from the Formal and Informal Meat Sectors in South Africa

**DOI:** 10.3390/ijerph15040819

**Published:** 2018-04-21

**Authors:** Ishmael Festus Jaja, Ezekiel Green, Voster Muchenje

**Affiliations:** 1 Department of Livestock and Pasture Science, University of Fort Hare, Alice 5700, South Africa; vmuchenje@ufh.ac.za; 2 Department of Biotechnology and Food Science, Faculty of Science, University of Johannesburg, Doornfontein 2028, South Africa; Egreen@uj.ac.za

**Keywords:** abattoir, *Escherichia coli*, food safety, meat, South Africa

## Abstract

Foodborne disease (FBD) is a global public health concern, and foods from animal sources have been associated with outbreaks of food-related illness. In this study, animal carcasses from the two abattoirs (HT1 and HT2) in the formal meat sector (FMS) and slaughter points in the informal meat sector (INMS) were examined at two stages of slaughter (before washing and after washing) for aerobic colony counts (ACC) and total viable count (TCC), as well as *Escherichia coli* and *Staphylococcus aureus* count. At each stage, carcasses were sampled by swabbing at the neck, brisket, flank, and rump. ACC for beef, mutton, and pork carcasses at HT1 and HT2 before washing were between 2.5–5.8, 2.2–4.7, and 2.7–3.7 mean log CFU/cm^2^, respectively, and TCC count before washing was highest on the neck of cattle (6.3 ± 2.4) and after washing was highest on the perineal of sheep (5.7 ± 6.9). In the INMS, TCC count was highest on the brisket (6.9 ± 3.2) and in the neck (5.5 ± 2.4). Higher ACC values of 6.2–6.7 mean log CFU/cm^2^ were obtained in the INMS. The highest count for *E. coli* (4.2 mean log CFU/cm^2^) after washing was in the neck, while the highest count for *S. aureus* (4.0 mean log CFU/cm^2^) was in the flank. All bacteria count in the INMS exceeded acceptable limits, and washing did not significantly reduce microbial load in meat in the FMS and INMS. Bacteria count in the FMS and INMS exceeded acceptable standards. However, meat processed in the INMS poses a more significant risk of FBD to consumers.

## 1. Introduction

Meat is a staple commodity in South Africa, consumed at a rate of 41.0 kg per capita per year. The nutritional composition of meat includes protein, fat, vitamins, and minerals such as iron, zinc, and phosphorus. Despite these benefits, meat has been well-known as a potential channel for spreading food-borne diseases due to its high water activity, high protein content, and approximately neutral pH, which create favourable conditions for the multiplication and survival of bacteria [1,2,3,4,5,6].

Although muscles of healthy animals do not contain microorganisms, meat tissues get contaminated during the various stages of slaughter and transportation. The risk of contamination happens from the point of entry of animals into the slaughters up to the time of meat consumption. In this regard, the abattoir environments and slaughter processes play leading roles in the spreading of microbial contamination [7,8]. Many factors may contribute as sources of contamination of carcasses along the chain of slaughter, including the animal’s skin and dung, equipment such as machines and cutting tools, an unhygienic environment, non-compliance with proper slaughter processes, and a lack of personal hygiene [8].

*Escherichia coli* and *Staphylococcus aureus* are among many common bacteria found to compromise meat quality. Many *E. coli* strains have emerged as important zoonotic foodborne pathogens. [9]. Hence, due to their ability to cause numerous sporadic cases and foodborne disease outbreaks in humans, they have become a significant public health threat [10,11,12]. *Staphylococcus aureus*, on the other hand, produces a variety of potent staphylococcal enterotoxins (SEs), which are resistant to inactivation by gastrointestinal proteases and are consequently responsible for staphylococcal food poisoning [10,13]. Severe gastroenteritis can develop within one to seven hours after the consumption of *Staphylococcus aureus* contaminated food, leading to diarrhoea, vomiting, and dehydration. Infections of the skin, soft tissue, joint, bone, respiratory, and endovascular disorders have frequently been reported [14]. Further complications such as pneumonia, meningitis, osteomyelitis, and toxic shock syndrome have been associated with staphylococcal infection [15].

In many developing countries, foodborne diseases (FDs) remain serious public health problems [12,16]. In South Africa, meat processed in the formal sector undergoes various checks for microbial quality. However, meat supplied from the informal sector is nonetheless not checked. The situation presents an opportunity for the supply of poor quality and contaminated meat products, enhancing the possibility of FD outbreak. A recent study indicates a high level of microbial contamination of beef carcasses at a high throughput abattoir in South Africa [17]. However, research about the quality of meat in the informal meat sector is limited. Thus, there is a need to determine the microbial quality of meat sold in the informal market. Hence, this study aims to assess the aerobic bacteria, coliform, *Escherichia coli,* and *Staphylococcus aureus* counts of raw meat, in the formal and informal meat sector.

## 2. Materials and Methods

### 2.1. Sample Collection

**Figure 1:**
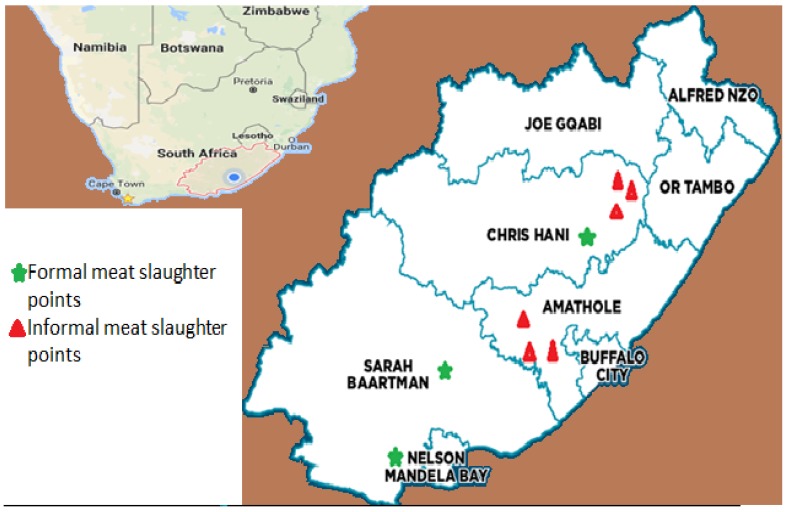
Map of the Eastern Cape Province showing sampling points.

A convenient sampling procedure was carried out using the simple random sampling method. The convenient method allowed sampling units to be selected at random based on the number of animals slaughtered on sampling days. All samples were collected between July and November 2015 from two high through-put abattoirs (HT1 and HT2) in the formal meat sector (FMS) and five animal slaughter points in the informal meat sector (INMS) (Figure 1). A total of 400 swabbed samples were collected using a sterile cotton throat swab from the HT1 (40 cattle) and HT2 (40 sheep and 20 pigs). In total, 112 swab samples were collected from the informal meat sector (15 cattle and 13 sheep). Four parts of each animal were sampled for cattle (rump, flank, brisket, and neck), sheep (perineum, flank, brisket, and neck), and pig (ham, back, belly, and jowl) [18,19]. A swab sample of the carcasses was performed after animal stunning, skinning, and evisceration. Swabbing was done before and after washing of the carcass. All swab samples were transported to the laboratory in a cooler box stocked with ice packs and processed within 3–8 h after collection.

### 2.2. Isolation and Identification of Organisms

Cotton throat swabs were replaced in sterile test tubes containing 0.8% saline solution, and vortexed for 2 min [19,20]. Serial decimal dilutions of the resultant suspension were prepared and 1 mL of each dilution was seeded in duplicate using the pour plate method onto dishes containing MacConkey agar (MCA) and plate count agar (PCA) (Oxoid) for coliform and total viable count, respectively, and incubated at 30 °C for 24–48 h [10]. *Escherichia coli* enumeration was repeated on Eosin methylene blue agar (Oxoid, Basingstoke, UK) by plating an appropriate dilution on plates followed by aerobic incubation at 37 °C for 24 h. After incubation, *E. coli* were counted as colonies with a distinct green metallic sheen [21,22]. For the counting and identification of *S. aureus*, 1 mL of diluent was seeded in duplicate using the pour plate method onto dishes containing a Mannitol salt agar plate (Oxoid) and incubated at 37 °C for 24 h. The microbial organisms that show a characteristic colony morphology of *S. aureus* on Mannitol salt agar (MSA) (Biolab, Midrand, South Africa) as a yellow coloured sheen were confirmed to be *S. aureus*. Mannitol fermentation and salt tolerance properties of *S. aureus* produced the typical yellow colonies because of the change in the pH [23]. Further identification of *S. aureus* was done using the Gram staining method, and standard biochemical assays such as oxidase and catalase tests. Gram staining was used to ascertain that there were no other airborne bacterial contaminants by confirming the characteristic morphology of *S. aureus*. Presumptive isolates of *E. coli* and *S. aureus* were stored in 20% glycerol stock pending further use.

All bacteria counts were done according to the standard prescribed by ISO 4833 and ISO 21528-2, and the result of bacteria enumeration was benchmarked with the South African standard for the microbiological monitoring of meat, process hygiene, and cleaning (VPN 15) (NDVQPH, 2010). As per VPN 15, aerobic plate counts are (i) acceptable (a): if counts are ≤3162 CFU/cm^2^ (3.5 log); (ii) marginal (m): if counts are ≤100,000 cfu/cm^2^ (5.0 log); and (iii) unacceptable (u): if counts are >100,000 cfu/cm^2^ (5.0 log). The VPN 15 regulation for *E. coli* count are as follows: (i) acceptable (a): if counts are ≤1 CFU/cm^2^ (0 log); (ii) marginal (m): if counts are ≤10 CFU/cm^2^ (1 log); and (iii) unacceptable (u): if counts are >10 CFU/cm^2^ (1 log) [24]. The VPN 15 document did not specify any limits to total coliform count (TCC) and *Staphylococcus aureus* count; hence the limits set for ACC and *E. coli* were adopted for TCC and *Staphylococcus aureus*, respectively.

### 2.3. Bacterial DNA Extraction

All isolates previously stored in glycerol stocks were resuscitated by inoculation into nutrient broth (Merck, Johannesburg, South Africa), and incubated at 37 °C for 24 h. The extraction of DNA was performed using a boiling method as previously described elsewhere [25]. Briefly, about three to five colonies were picked with the aid of a sterile wire loop and put into sterile DNAase/RNAase-free Eppendorf tubes (Biologix, Lenexa, KS, USA) containing 200 μL nuclease-free water (Thermo Scientific, Waltham, MA, USA). Each suspension was mixed using a vortex machine (Digisystem Laboratory, New Taipei, Taiwan), and the cells were lysed by heating for 15 min in a heater block (Lasec, Cape Town, South Africa) at 100 °C. The lysate was then centrifuged at 13,000 rpm for 5 min, and the supernatant was collected in a sterile Eppendorf tube and stored at −20 °C for further use.

### 2.4. Molecular Identification of S. aureus and E. coli Isolates

A standard polymerase chain reaction (PCR) for species-specific thermonuclease (*Nuc*) gene amplification was performed to detect *S. aureus* strains from the samples, and *UidA* was used for detecting *E. coli*. Isolates were confirmed to be positive for *S. aureus* if they were defined as Gram-positive, catalase-positive cocci showing a positive PCR result for the *Nuc* gene, which is highly specific for *S. aureus* [26]. A confirmatory PCR mix was in a total volume of 25 µL containing 5.0 µL of the DNA template, 5.5 µL nuclease-free water, 12.5 µL master mix, 1.0 µL forward primer, and 1.0 µL reverse primer. *Escherichia coli* (ATCC 25922) and *Staphylococcus aureus* (ATCC 25923) served as the positive control strains in each test protocol [25,27,28,29]. The PCR conditions for *Escherichia coli* and *Staphylococcus aureus* are listed in Table 1.

All reactions were carried out in a MyCyclerTM Thermal Cycler PCR system (BioRad, Hercules, CA, USA). The PCR products (5 μL) were subjected to 1.5% agarose gel electrophoresis (Separations, South Africa) stained with 0.001 μg/mL ethidium bromide (Sigma-Aldrich, St. Louis, MO, USA) using 0.5× Tris-borate EDTA (TBE) buffer at 100 V for 60 min. The gel was visualized under the UV transilluminator (Alliance 4.7, UVItec, Cambridge, UK). A 100 bp DNA ladder (Promega, Madison, WI, USA) was used as the molecular size standard for expected band size.

### 2.5. Statistical Analysis

All data analysis was performed using Microsoft^®^ Excel version 2007, (Microsoft, Redmond, WA, USA) mathematical functions and Statistical Package for Social Sciences (SPSS) version 22 (SPSS Inc., Chicago, IL, USA). Descriptive statistics were used to express the mean log, and standard deviation of the *E. coli* counts from the neck, brisket, flank, and rump of carcasses, before and after washing with water. A paired sample *t*-test was used to determine the difference in means before and after washing, and a value of *p* < 0.05 was considered significant.

### 2.6. Ethical Approval

Ethical clearance number MUC351SJAJ01 was obtained from the University of Fort Hare ethics committee before the commencement of sample collection, and approval to collect samples from the abattoirs was also obtained from the abattoirs.

## 3. Results

### 3.1. Aerobic Colony Counts and Total Coliform Count

A marginal decrease was observed for aerobic colony counts (ACC), total coliform count (TCC), and *E. coli* and *Staphylococcal* count after carcass washing in the HT1 and HT2. However, notwithstanding carcass washing in HT1, ACC for jowl increased by 0.8 mean log CFU/cm^2^ (Table 2). The enumeration of ACC before carcass washing at HT1 ranged from 2.5 to 5.8 mean log CFU/cm^2^; after washing, the count ranged from 2.1 to 4.3 CFU/cm^2^. Similarly, a negligible decrease in ACC from 6.2–6.7 to 5.1–5.2 mean log CFU/cm^2^ was observed in the informal sector after carcass washing (Table 3). The TCC before carcass washing in the formal meat sector (FMS) ranged from 5.0 to 6.3 mean log CFU/cm^2^, whereas after washing, the counts ranged from 4.6 to 6.3 CFU/cm^2^.

The VPN 15 regulations for aerobic plate count and total coliform count are (i) acceptable (a): if counts are ≤3162 cfu/cm^2^ (3.5 log); (ii) marginal (m): if counts are ≤100,000 cfu/cm^2^ (≤5.0 log); and (iii) unacceptable (u): if counts are >100,000 cfu/cm^2^ (>5.0 log). 

The VPN 15 regulations for *E. coli* count are as follows: (i) acceptable (a): if counts are ≤1 cfu/cm^2^ (0 log); (ii) marginal (m): if counts are ≤10 cfu/cm^2^ (1 log); and (iii) unacceptable (u): if counts are >10 cfu/cm^2^ (1 log) (NDVQPH 2010).

The VPN 15 regulations for aerobic plate count and total coliform counts are: (i) acceptable (a): if counts are ≤3162 cfu/cm^2^ (3.5 log); (ii) marginal (m): if counts are ≤100,000 cfu/cm^2^ (≤5.0 log); and (iii) unacceptable (u): if counts are >100,000 cfu/cm^2^ (>5.0 log).

The VPN 15 regulations for *E. coli* count are as follows: (i) acceptable (a): if counts are ≤1 cfu/cm^2^ (0 log); (ii) marginal (m): if counts are ≤10 cfu/cm^2^ (1 log); and (iii) unacceptable (u): if counts are >10 cfu/cm^2^ (1 log) (NDVQPH 2010).

INMS ranged from 5.3 to 5.4 mean log CFU/cm^2^. However, washing led to a 0.2 mean log CFU/cm^2^ increase in the TCC for perineum.

## 3.2. Escherichia coli and Staphylococcus aureus Count

Washing led to a minimal decrease in the *E. coli* count in cattle at HT1, except for sheep flank and briskets in HT2, where the count increase by 0.1 and 0.6 mean log CFU/cm^2^, respectively. Likewise, the count for pig ham and belly increased by 0.5 and 1.0 mean log CFU/cm^2^, respectively (Table 2). The *E. coli* enumeration in the INMS was higher than the standard value for red meat and washing only minimally reduced the count in cattle. However, the count for sheep brisket and neck increased by 0.5 and 1.0 mean log CFU/cm^2^ after washing, respectively (Table 3). *Staphylococcus aureus* count for the formal meat sector (HT1 and HT2) did not significantly (*p* < 0.05) differ after carcass washing. The *S. aureus* count for pig belly increased by 0.4 mean log CFU/cm^2^. The *S. aureus* count in the INMS was similar to those obtained from the FMS. The molecular detection of *S. aureus* in beef, mutton, and pork carcasses at HT1 and HT2 was 16.5%, 15.7%, and 9%, respectively. Whereas 3.6%, 7.9%, and 15% *S. aureus* was detected in slaughter mens’ hands at HT1, HT2, and INMS, respectively (Table 4).

## 4. Discussion

The majority of poor people in low and middle-income countries purchase meat from the informal market because the meat is cheap, and the market is often proximal to rural communities [30,31]. However, in the absence of meat safety standards and hygiene, the chemical composition of meat favours microbial growth to unacceptable levels. Hence, microbially contaminated meat poses the risk of transmission of foodborne diseases (FBD) to consumers. The prevalence of FBD is a growing global problem, especially in developing countries, where hygiene management systems are poorly implemented [19,32,33,34]. Since major gut-dwelling foodborne pathogens such as *Salmonella*, *E. coli*, *Campylobacter*, and other enterobacteria are excreted from the gastrointestinal tract of food-producing animals, cross-contamination is often a result of poor slaughter technique and hygiene standards at abattoirs [11,35,36,37,38]. Sampling for Microbial estimation in the rump, flank, brisket, and neck have been conducted in many countries including Namibia, Ireland, Serbia, and Switzerland [19,39,40,41], but to our knowledge, none have been conducted in the Eastern Cape Province, South Africa. Furthermore, the 2017–2018 outbreak of listeriosis has been linked to the contamination of meat packages, justifying the necessity of continuous sampling of various parts of carcasses, packaging, and abattoir environments.

The results for FMS show that the rump, neck, perineum, ham, and jowl were the most contaminated. However, in the INMS, contamination was not specific to any part of the carcass, as almost all parts of the carcass were contaminated. On some occasions, animal carcasses were manually pulled or push along the slaughter rail. Hence this could account for the level of contamination reported for the rump, neck, perineum, ham, and jowl. In similar studies, the brisket yielded a higher result that the neck, flank, and rump [19,42], but in another study, microbial contamination did not significantly differ for the rump, flank, brisket, and neck [43].

Washing of carcasses in the informal sector did not significantly (*p* < 0.05) decrease the level of microorganism in meat and probably contributed to further spread of the contamination of carcass. Although the bacteria enumeration in carcasses in the formal sector (FMS) yielded fewer counts when compared to those from the informal meat sector (INMS), bacterial levels, however, in many instances, exceeded the regulated benchmark [24]. For example, the TCC count at HT1 for the neck (4.6 ± 2.5) and rump (5.4 ± 3.1) mean log CFU/cm^2^ even after washing is worrisome. Likewise, the TCC count at HT2 of the perineum (5.7 ± 6.9 mean log CFU/cm^2^) after washing is concerning. Washing in these instances led to an increased microbial count in meat. Similarly, *E. coli* and *S. aureus* count in FMS and INMS exceeded acceptable limits even after washing. 

The result of this study strongly suggests some basic hygiene problems along the slaughter and processing chains, as ACC, TCC, and *E. coli* counts are standard methods for estimating the microbial contamination of carcasses [1,7,8,19]. In one study of *Salmonella*, *Escherichia coli*, Enterobacteriaceae, and aerobic colony count, during beef slaughter in the Eastern Cape Province of South Africa, washing did not reduce the microbial load in the carcass [17]. Studies elsewhere reported a similar finding that washing did not reduce the microbial contamination of meat [18,20,21]. However, in many of the studies, a significant reduction in bacteria counts was only noted after the singeing, scalding, blasting, and chilling of meat [17,19,20].

Meat from the INMS seldom undergoes blasting and chilling, which underscores the high risk of FBD associated with the consumption of such meat. The purchase and consumption of meat from such sources have further been linked to increased risk of food poisoning [38,44,45]. The high bacteria count detected in the meat from INMS depicts the unregulated nature of slaughter processes and marketing in the sector. The absence of refrigeration, sanitation, adequate health inspection, and risk analysis is responsible for the foodborne illness associated with the consumption of meat from the INMS [46]. Process stages such as stunning, skinning, scalding, evisceration, and chilling are sensitive points where microbial contamination of carcass can be prevented. Hence establishing a critical control point along this chain is essential [18,36,47,48].

Foodborne pathogens such as *E. coli* and other *enterobacteria* are essential causes of diarrhoeal diseases. Enteric and diarrheal diseases are important causes of childhood death in the low-and-middle-income countries; and are regarded as the second cause of death, after lower respiratory diseases in children under five years old [49,50,51]. The South African National Institute for Communicable Diseases (NCID) report of foodborne disease (FBD) for January to June 2017 indicated that *Staphylococcus*, *Salmonella,* and *Clostridium perfringens* were the leading cause of FBD in the period previously mentioned [52]. Hence, the result of the present study highlights, once more, the importance of proper cleaning and disinfection procedures in the process control at abattoirs during slaughter. It also exposes the lax in good manufacturing practice and hygiene management and critical control points (HACCP) at the slaughterhouses.

The prevalence of *S. aureus* in the FMS (22.7%, and 33.7%) and INMS (68%) can be compared to the prevalence of *S. aureus* elsewhere [1,22,53,54,55]. However, a lower prevalence of *S. aureus* was reported in studies conducted in Brazil and Pakistan [7,56]. *Staphylococcus aureus* can asymptomatically survive in the skin and nasal vestibules of humans and animals. Roughly 20% of humans are considered as persistent carriers, and 30% are intermittent carriers of *S. aureus* in the nostrils [33,57].

Many slaughter workers were observed during sample collection to be singing during slaughter, enhancing the possibility of oral to carcass contamination at such times. Hence the prevalence of *S. aureus* in this study would be expected because it is a principal component of the nasal vestibule and skin. Although *S. aureus* commensally inhabits the skin and nasal passage, it can cause widespread infections, from superficial skin infections to severe, and potentially fatal, invasive disease, including food-related illness [58]. Staphylococcal food poisoning triggered by enterotoxin-producing *S. aureus* is a significant foodborne disease globally [59]. In many countries, the foods that most often cause this type of food poisoning are red meat and their products [53].

It is reasonable to think that apart from cross contamination by abattoir workers, other sources of contamination could be dirty hides, abattoir environments, knives, and other cutting machines [36,41,60,61]. Stricter process hygiene control is necessary to improve the microbial quality of meat. The limitation of the present study would be that the study compared the microbial quality of meat in only two high throughput abattoirs and meat slaughter points in the informal market. The result obtained from the study should not be generalised for all formal abattoirs in the province. Furthermore, some selective media could hamper the multiplication of cells with sub-lethal injuries, thereby hindering their growth and ultimately affecting the counts. However, these limitations did not diminish the important finding in the study. Complete eradication of microbial contamination of meat may be very difficult to achieve, but microbial counts above an acceptable level present a problem not only to the quality and shelf life of meat but also to the consumer. Since washing did not substantially reduce the microbial load, the study buttresses the need for the adoption of pre-treatment of animal hide and skin, as well as carcasses, as practised in many countries [19,62,63] and strict adherence to hygiene management and assessment system.

## 5. Conclusions

Microbial count of meat in the formal and informal meat sectors exceeded acceptable limits by South African regulation. The high ACC, TCC, *E. coli*, and *S. aureus* counts in meat from the formal and informal meat sectors not only point to the existence of contamination and a weak hygiene management system, but also highlight the high risk of consumers contracting meat-related infections if not properly cooked. Foodborne diseases remain a global public health crisis which requires multifaceted, integrated, regional, and international collaborative solutions. The problem of meat hygiene in the informal setting could be even worse given the ubiquitous and unstructured nature of the sector. Interventions aimed at educating communal farmers who are the primary suppliers of meat in the INMS, consumers, and meat handlers could lead to an improved meat hygiene culture. Moreover, the training and re-training of slaughter workers on good slaughter practices (GSPs) and a hygiene management system (HMS) are crucial.

## Figures and Tables

**Table 1:** Primers and PCR conditions for molecular identification of *S. aureus.*

Gene	Sequence	Product Size (bp)	PCR Conditions	References
*Nuc* gene	F 5′-GCGATTGATGGTGATACGGTT-3′	270	Initial denaturation at 95 °C for 5 min was followed by 37 cycles of amplification (denaturation at 95 °C for 30 s, annealing at 55 °C for 30 s, and extension at 72 °C for 60 s) and ending with a final extension at 72 °C for 10 min.	[27,28]
	R 5′-AGCCAAGCCTTGAACGAACTAAAGC-3′			
*UidA* gene	F 5′AAAACGGCAAGAAAAAGCAG-3′	147	Initial denaturation at 94 °C for 2 min followed by 25 cycles of denaturation at 94 °C for 1 min, annealing at 58 °C for 1 min, and extension at 72 °C for 1 min, and ended with a final extension at 72 °C for 2 min. Holding was at 4 °C.	[29]
	R 5′ACGCGTGGTTAACAGTCTTGCG-3′			

**Table 2:** The logarithm_10_ of mean total bacterial counts for cattle meat samples from selected abattoirs in the Eastern Cape Province, South Africa.

Meat Sector	Specie	Sampling Point	Number of Carcasses	Log_10_ Mean Total Bacterial Counts ± SD (log_10_ cfu/cm^2^)							
				ACC		TCC		*E. coli*		*S. aureus*	
			*n*	BW	AW	BW	AW	BW	AW	BW	AW
HT1	cattle	Rump	40	4.2 ± 2.2	2.7 ± 1.6	5.0 ± 2.1	4.6 ± 2.5	4.1 ± 2.1	3.2 ± 2.0	3.9 ± 2.4	3.2 ± 2.4
		Flank	40	2.5 ± 1.5	2.1 ± 1.3	4.1 ± 2.4	3.8 ± 2.5	3.6 ± 2.8	2.9 ± 1.9	4.2 ± 2.6	3.8 ± 2.4
		Brisket	40	2.7 ± 1.5	2.6 ± 1.3	4.3 ± 2.7	4.3 ± 2.7	3.7 ± 2.2	3.3 ± 1.9	3.7 ± 2.2	2.8 ± 1.8
		Neck	40	5.8 ± 2.6	4.3 ± 2.5	6.3 ± 2.4	5.4 ± 3.1	5.0 ± 2.7	4.2 ± 2.6	3.9 ± 2.3	3.6 ± 2.3
HT2	Sheep	Perineal	40	3.2 ± 2.4	2.9 ± 2.4	4.6 ± 2.5	5.7 ± 6.9	2.8 ± 2.6	2.8 ± 2.5	4.2 ± 2.6	3.4 ± 2.2
		Flank	40	2.4 ± 1.6	2.3 ± 1.7	3.5 ± 2.4	3.5 ± 2.3	2.5 ± 2.0	2.6 ± 1.9	4.9 ± 2.7	4.0 ± 2.5
		Brisket	40	2.2 ± 1.5	2.3 ± 1.8	3.9 ± 2.3	3.6 ± 2.2	2.1 ± 1.6	2.7 ± 2.2	4.1 ± 2.4	2.9 ± 1.7
		Neck	40	4.7 ± 2.7	4.3 ± 2.5	4.5 ± 2.6	4.2 ± 2.8	3.5 ± 2.3	3.2 ± 2.0	3.9 ± 1.9	3.7 ± 2.3
	Pig	Ham	20	3.6 ± 1.8	3.5 ± 2.7	4.8 ± 2.4	3.2 ± 1.9	2.5 ± 1.5	3.0 ± 1.7	3.5 ± 1.5	2.7 ± 1.5
		Back	20	2.7 ± 1.0	2.7 ± 0.9	3.1 ± 0.9	1.1 ± 1.0	3.7 ± 2.0	2.9 ± 1.1	4.3 ± 2.3	3.2 ± 1.7
		Belly	20	2.9 ± 1.1	2.7 ± 1.5	3.9 ± 2.2	3.0 ± 1.5	2.6 ± 1.4	3.6 ± 1.6	2.9 ± 1.2	3.3 ± 1.5
		Jowl	20	3.7 ± 1.3	4.5 ± 1.9	4.2 ± 2.0	2.8 ± 2.1	2.6 ± 1.7	2.7 ± 1.1	5.3 ± 2.0	3.2 ± 1.6

HT1 and HT2: High throughput abattoirs in the formal meat sector; *n*: Number of animals sampled; ACC: Aerobic colony count; TCC: Total coliform count; SD: Standard deviation; BW: Before washing; AW: After washing.

**Table 3:** The logarithm_10_ of mean total bacterial counts for cattle meat samples from selected location in the informal meat sector in the Eastern Cape Province, South Africa.

Meat Sector	Specie	Sampling Point	Number of Carcasses	Log_10_ Mean Total Bacterial Counts ± SD (log_10_ cfu/cm^2^)							
				ACC		TCC		*E. coli*		*S. aureus*	
				BW	AW	BW	AW	BW	AW	BW	AW
INMS	Cattle	Rump	15	6.4 ± 3.6	5.2 ± 2.5	5.4 ± 3.5	4.9 ± 2.6	6.5 ± 3.0	5.4 ± 2.7	5.1 ± 2.2	4.3 ± 1.3
		Flank	15	6.2 ± 3.1	4.8 ± 2.5	4.5 ± 2.0	3.7 ± 1.9	4.7 ± 2.3	4.0 ± 3.0	5.6 ± 2.4	4.8 ± 2.0
		Brisket	15	6.6 ± 3.1	4.8 ± 3.0	6.9 ± 3.2	4.0 ± 2.2	5.3 ± 2.6	3.8 ± 2.3	5.2 ± 2.5	5.1 ± 2.4
		Neck	15	6.7 ± 3.5	5.1 ± 3.3	5.3 ± 3.1	5.5 ± 2.4	5.0 ± 2.6	4.3 ± 2.1	5.3 ± 2.5	4.9 ± 1.4
INMS	Sheep	Perineal	13	4.4 ± 3.4	4.1 ± 2.8	4.7 ± 3.0	3.8 ± 2.3	6.3 ± 3.5	5.7 ± 2.4	4.6 ± 1.9	4.2 ± 1.3
		Flank	13	3.2 ± 1.5	2.8 ± 1.2	4.7 ± 2.2	4.3 ± 1.8	4.0 ± 2.3	3.0 ± 1.6	5.7 ± 1.7	4.2 ± 1.5
		Brisket	13	4.7 ± 1.3	4.0 ± 0.9	5.6 ± 2.8	3.6 ± 1.7	4.5 ± 2.7	5.0 ± 2.7	4.8 ± 2.7	4.7 ± 2.1
		Neck	13	4.4 ± 1.1	3.7 ± 0.8	4.8 ± 1.5	4.8 ± 1.5	4.8 ± 2.2	5.8 ± 2.9	4.5 ± 2.2	4.5 ± 0.9

INMS: samples obtained from the informal meat sector; ACC: Aerobic colony count; TCC: Total coliform count; SD: Standard deviation; BW: Before washing; AW: After washing.

**Table 4:** Presence of *S. aureus* isolated from two high throughout abattoirs [HT1 (*n* = 194) and HT2 (*n* = 89)] and informal meat sector [INMS (*n* = 100)].

Abattoirs	Sampling Points	Sample Size	Presumptive Isolates (%)	Confirmed with PCR (%)
HT1	Cattle	168	109 (56.2)	32 (16.5)
	slaughtermen	12	12 (6.2)	7 (3.6)
	Door handle	4	3 (1.5)	0 (0)
	Knife	8	7 (3.6)	5 (2.6)
	Saw	2	1 (0.5)	0 (0)
	Total	194	132 (68)	44 (22.7)
HT2	Sheep	36	27 (30.3)	14 (15.7)
	Pig	36	21 (23.6)	8 (9)
	slaughtermen	8	8 (9)	7 (7.9)
	Door handle	2	1 (1.1)	0 (0)
	Knife	5	3 (3.4)	1 (1.1)
	Saw	2	0 (0)	0 (0)
	Total	89	60 (67.4)	30 (33.7)
INF	Cattle	20	16 (16)	16 (16)
	Sheep	52	44 (44)	32 (32)
	Slaughter men	16	15 (15)	15 (15)
	Knife	12	8 (8)	5 (5)
	Total	100	83 (83)	68 (68)

HT1 and HT2: High throughput abattoir in the formal meat sector (FMS), INMS: Informal meat sector.
